# Intimate partner violence against women and the adoption of unhealthy weight control practices

**DOI:** 10.1590/1980-549720250048

**Published:** 2025-09-19

**Authors:** Marina Rodrigues de Almeida, Claudia Leite de Moraes, Maria Helena Hasselmann, Michael Eduardo Reichenheim, Emanuele Souza Marques

**Affiliations:** I Universidade Estadual do Rio de Janeiro, Institute of Social Medicine Hesio Cordeiro, Department of Epidemiology - Rio de Janeiro (RJ), Brazil.; II Universidade Estácio de Sá, Medical School - Rio de Janeiro (RJ), Brazil.; III Universidade Estadual do Rio de Janeiro, Institute of Nutrition - Rio de Janeiro (RJ), Brazil.

**Keywords:** Intimate partner violence, Exposure to violence, Vomiting, Laxatives, Fasting, Women, Violência por parceiro íntimo, Exposição à violência, Vômito, Laxantes, Jejum, Mulheres

## Abstract

**Objective:**

To investigate the relationship between intimate partner violence (IPV) against women and unhealthy weight control practices (UWCP).

**Methods:**

This cross-sectional study was conducted in the metropolitan area of Rio de Janeiro, Brazil. It involved a sample of 847 women aged over 18 years, selected through a probabilistic complex sampling method. The Revised Conflict Tactics Scales were employed to assess psychological and physical IPV, while a single question was used to identify UWCP, namely, whether the respondent had ever used laxatives, diuretics, or vomiting to eliminate excess food, or had not eaten or eaten very little food to lose weight or not gain weight. Multivariate logistic and multinomial regression were employed to examine the relationship between exposure to IPV and the outcomes.

**Results:**

The findings indicated that exposure to any form of IPV increases the likelihood of self-induced vomiting, particularly among those exposed to physical IPV (odds ratio [OR] 1.75; 95% confidence interval [CI] 1.42-2.16). Additionally, physical IPV increases the odds of skipping meals (OR 1.28; 95%CI 1.05-1.57).

**Conclusion:**

It is therefore recommended that health professionals be aware of this relationship and consider the possibility that patients with eating disorders may be victims of IPV. This will ensure that their treatment and approach are comprehensive and targeted for more effective care.

## RESUMO

**Objetivo:**

Investigar a relação entre a violência entre parceiros íntimos (VPI) contra a mulher e as práticas não saudáveis de controle de peso (PCNP).

**Métodos:**

Trata-se de um estudo transversal realizado na área metropolitana do Rio de Janeiro, Brasil. A amostra incluiu 847 mulheres com mais de 18 anos, selecionadas por meio de um método de amostragem probabilística complexa. As escalas *Revised Conflict Tactics Scales* (CTS-2) foram utilizadas para avaliar a VPI psicológica e física, enquanto uma pergunta única foi usada para identificar cada PCNP - se a entrevistada já havia usado laxantes, diuréticos ou vômitos para eliminar o excesso de comida, ou se não havia comido ou comido muito pouco para perder ou não ganhar peso. Regressão logística e multinomial foram empregadas para examinar a relação entre a exposição à VPI e os desfechos de interesse.

**Resultados:**

Os achados mostraram que a exposição a qualquer forma de VPI aumentou a chance de vômito autoprovocado, principalmente entre aquelas expostas à VPI do tipo física (*odds ratio* - OR 1,75; intervalo de confiança de 95% - IC95% 1,42-2,16). Além disso, a VPI física aumentou a probabilidade de pular refeições (OR 1,28; IC95% 1,05-1,57).

**Conclusão:**

O estudo reforça a importância de os profissionais de saúde estarem cientes dessa possível relação e considerarem a possibilidade de que pacientes com sintomas de transtornos alimentares possam ser vítimas de VPI. Tal conduta poderá garantir o tratamento e abordagem mais abrangentes e direcionados para um atendimento mais eficaz.

## INTRODUCTION

Intimate partner violence (IPV) against women constitutes a violation of human rights^1^ and public health concerns^2^ - recognized by the World Health Organization - and is included in the United Nations’ 2030 Agenda (Sustainable Development Goal 5), specifically targeting the elimination of all forms of violence against women perpetrated by intimate partners^3^.

IPV is defined as any behavior that causes physical, sexual, or psychological harm, including acts of physical aggression, sexual coercion, psychological abuse, and controlling behaviors committed in a relationship between intimate partners, regardless of cohabitation or formal relationship^2^ ^,^ ^4^. Global estimates show that approximately one-third of women over the age of 15 have experienced physical and sexual IPV in their lives^4^ ^,^ ^5^ ^,^ ^6^.

The repercussions of exposure to IPV include chronic pain, physical injury (such as abrasions and fractures), gastrointestinal problems, and hypertension^7^, as well as sexual and reproductive health problems^7^ ^,^ ^8^. In addition to physical consequences, IPV is also associated with mental health problems, including symptoms of depression^7^ ^,^ ^8^ ^,^ ^9^ ^,^ ^10^, post-traumatic stress disorder^5^ ^,^ ^7^ ^,^ ^8^, and suicidal ideation^8^.

In recent decades, the literature has explored the relationship between IPV and eating-related outcomes, including both clinically diagnosed eating disorders (EDs) - named complete EDs - and Unhealthy Weight Control Practices (UWCP), also referred to as disordered eating behaviors^11^ ^,^ ^12^ ^,^ ^13^ ^,^ ^14^ ^,^ ^15^. UWCP refers to strategies for weight control - such as food restriction, binge eating, and purging (via self-induced vomiting or use of laxatives and diuretics) - that do not necessarily meet the frequency, intensity, or duration criteria required for the clinical diagnosis of an ED^16^ ^,^ ^17^. These behaviors, while not classified as classic EDs, are nevertheless considered harmful due to their physical and psychological consequences and may represent early markers or subclinical expressions of disordered eating.

Some studies have highlighted the relationship between IPV exposure and engagement in UWCP among women. Silverman et al.^11^ identified this link among female adolescents in the USA. Neumark-Stainer et al.^12^ reported a significantly increased risk of laxative use and self-induced vomiting among American adolescents exposed to IPV. Bonomi et al.^13^ found that female university students in the USA with a history of physical and/or sexual IPV were more likely to engage in weight control strategies such as diet drug use and inducing vomiting. More recently, Yoon et al.^15^ associated binge eating with experiences of sexual and physical abuse perpetrated by an intimate partner in American students. It is important to note that studies are still few and restricted to the United States, highlighting the limited evidence on the association between IPV and UWCP in low- and middle-income countries.

In addition, some studies have examined the cumulative impact of exposure to multiple forms of violence on disordered eating behaviors. The studies suggest a greater risk of engaging in UWCP (binge eating, self-induced vomiting, use of laxatives or diuretics, and excessive weight monitoring) after exposure to episodes of violence. According to the authors, the likelihood of engaging in UWCP increases with the number of violent experiences reported^15^ ^,^ ^18^.

Although there is already some evidence on the subject, it is important to note that studies focusing on exposure to IPV and the development of disordered eating behaviors are recent, scarce, and limited to certain types of violence, mainly physical and sexual violence, especially in childhood. It should be noted that most of the studies did not evaluate UWCP separately^11^ ^,^ ^19^ and/or had only adolescents as the study population^11^ ^,^ ^12^ ^,^ ^13^ ^,^ ^15^ ^,^ ^19^ ^,^ ^20^.

Given that isolated disordered eating behaviors can evolve into full-blown classic disorders with the presence of compensatory behaviors^17^ ^,^ ^21^ ^,^ ^22^, evaluating the factors that lead to the adoption of such behaviors is important for identifying at-risk groups and guiding prevention and early intervention efforts. Thus, this study aimed to assess the relationship between psychological and physical violence against women by intimate partners and the adoption of unhealthy weight control practices in adult women.

## METHODS

### Local

This study is a household survey carried out in the municipality of Duque de Caxias, located in the Metropolitan Region of Rio de Janeiro, which covers an area of 467.3 km^2^. The 2022 Institute of Geography and Statistics (IBGE) Demographic Census indicates that the municipality has a population of 782,799 inhabitants. The municipality is predominantly urban. According to the data from the Brazilian IBGE, the municipality had a Human Development Index (HDI) of Duque de Caxias was 0.711 in 2010, below the national average of 0.760 for the same year^23^. In 2021, the per capita gross domestic product (GDP) was R$ 57,170^24^. Data from IBGE’s *New Poverty Map* indicate that 30.48% of the population in Duque de Caxias lives in poverty^25^. Compared to national averages, the municipality historically presents lower HDI and GDP per capita, along with higher poverty prevalence and a greater proportion of families living in poverty^23^ ^,^ ^24^ ^,^ ^25^.

### Study design, sampling, data collection, and participants

This is a population-based cross-sectional study developed in Duque de Caxias, Greater Metropolitan Rio de Janeiro, Brazil. Participants were selected using a three-stage complex sampling strategy, involving the selection of census districts, permanent private households, and individuals.

In the first stage, census districts within the District of Campos Elíseos were selected with probability proportional to the number of permanent private households. The districts were ordered by average household income to ensure representation across all income levels. In the second stage, 10 households were systematically selected within each sampled district, ensuring that each household had an equal probability of selection. Inverse sampling techniques, as described by Haldane^26^, were employed in the field to reach the target number of respondents. In the third stage, all eligible individuals within selected households were invited to participate.

Data collection occurred between April and November 2010. A face-to-face interview was conducted with the woman of reference in the household. The interview took place in a reserved area of the home, absence of the male partner. The modules about intimate issues were administered exclusively by female interviewers.

For this study, we included participants who met the following eligibility criteria:

1. Women over the age of 18; and
2. Self-reported involvement in a romantic or intimate relationship in the 12 months preceding the date of the interview.

Based on these criteria, the final sample consisted of 847 women, representing 81.8% of the original sample.

### Theoretical-operational model

**Figure 1.**
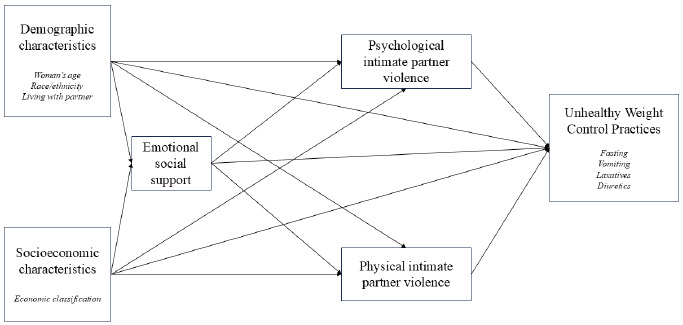
Theoretical-operational model representative of variables related to intimate partner violence against women and unhealthy weight control practices.

The theoretical-operational model (Figure 1) was developed based on a comprehensive review of the thematic literature, with IPV identified as the exposure and disordered eating behaviors as the outcome. The model illustrates the potential relationship between IPV and these behaviors without attempting to exhaust all dimensions associated with both phenomena. The following sections will provide a detailed account of the variables that constitute each dimension of the model, along with the methods used to measure them.

### Variables

#### Intimate partner violence against women: exposure

The IPV measure employed was the Portuguese version of the Revised CTS2, which was adapted for use in Brazil^27^ ^,^ ^28^.

The study period spanned 12 months preceding the interview. The physical violence subscale of the CTS2 comprises 12 items, while the psychological violence subscale contains eight items. Each item is rated on a frequency-based scale, with the following options: “never,” “once,” “twice,” “3-5 times,” “6-10 times,” and “more than 20 times.” The presence of at least one affirmative response among the dichotomous items on the respective subscale was used to define physical and psychological IPV. The variables were operationalized as scores for the causal analyses, whereby the total score for each subscale is the sum of the item responses.

#### Unhealthy Weight Control Practices (UWCP): outcome

The UWCP encompassed laxative and diuretic use, self-induced vomiting, and fasting. These practices were operationalized in two distinct ways. First, each practice was analyzed individually as a dichotomous variable based on participants’ self-reports in the previous 6 months.

1. Laxatives (drugs that cause diarrhea) to eliminate excess food ingested;
2. Diuretics (drugs that make you urinate a lot) to eliminate excess food intake;
3. Inducing vomiting to eliminate excess food with the intention of losing weight or not gaining weight;
4. Not eating or eating very little food to lose weight or not gain weight.

A positive response to any of the aforementioned questions was deemed indicative of the adoption of the corresponding practice.

Secondly, the UWCP were analyzed collectively using a categorical variable comprising three levels:

1. Those who did not adopt any practices;
2. Those who adopted only one practice; and
3. Those who adopted two or more practices.

#### Socio-economic and demographic variables

The Brazilian Economic Classification Criterion (CCEB) was employed as a proxy for socioeconomic status^29^. This variable was operationalized into three categories: A + B, C, and D + E. The variable of race or color was categorized as “white” and “black/mixed/yellow/indigenous” based on the interviewee’s self-declaration following the classification system adopted by the IBGE. The cohabitation variable was defined following the household situation of the woman interviewed, specifically whether she resides with the intimate partner referred to in the questionnaire. The age variable represents the woman’s age in years at the time of the interview.

#### Social support

Social support was measured using the Medical Outcomes Study (MOS)^30^ ^,^ ^31^. This multidimensional instrument was adapted and validated for use in Brazil^31^ ^,^ ^32^. This study included only the emotional support subscale that comprises four items:

If you need to, how often do you have someone

1. To listen to you when you need to talk?;
2. To confide in or talk to about your problems?;
3. To share your innermost worries and fears?; and
4. Who understands your problems?^32^

Response options are rated on a five-point Likert scale (1=never to 5=always), yielding a total score ranging from 4 to 20, whereby higher scores indicate superior emotional well-being. In this analysis, emotional support was operationalized as a continuous variable.

### Data analysis

For exploratory purposes, the distribution of each variable in the study population was initially assessed by absolute and relative frequencies and their respective 95% confidence intervals for categorical variables and mean and standard deviation (SD) for continuous variables. Subsequently, bivariate analyses were conducted to examine the relationships between the exposure variable (IPV) and the outcome variables (UWCP separately and together), irrespective of other covariates. To investigate this relationship, a multivariate logistic or multinomial regression was employed. The use of different types of regression models was guided by the way the outcome variable was operationalized in each analysis.

The selection of covariates for the multivariate analysis was guided by a theoretical-operational model (Figure 1) developed by the research team, based on prior literature and conceptual frameworks relevant to the causal hypothesis under investigation.

It is important to highlight that the dataset used had a low proportion of missing data for the variables included in the analysis, with rates ranging from 2.0 to 11.5% (Supplemental Material 1). Therefore, all analyses were performed using complete data, and no imputation procedures were applied.

Results are presented as coefficients with 95% confidence intervals, considering p<0.05 statistically significant. All analyses were conducted using the Stata 17.0 svy suite, which is designed to handle complex sampling designs. More details of the methodology were in Supplemental Material 2.

### Ethical aspects

The research project was approved by the Research Ethics Committee of the Institute of Collective Health Studies of the Federal University of Rio de Janeiro in 2009 (No. 73/2009). The participants were informed about the procedures to be carried out, guaranteed anonymity, and were given the option to refuse to take part in the study. They were then asked to sign a free and informed consent form.

### Data availability statement

The entire dataset supporting the findings of this study is available upon request to the corresponding author, Emanuele Souza Marques. The dataset is not publicly available due to containing information that compromises the privacy of research participants.

## RESULTS

As illustrated in Table 1, the study population consisted of 847 women aged between 18 and 64, with a mean age of 39.9 years (SD: 12.0). The majority of the women were classified in economic stratum C (67.2%), self-declared as non-white (63.4%), and nearly 90% resided in the same household as their partner. The prevalence of psychological and physical IPV, as reported by the women in the study, was 60.9% (n=526) and 16.8% (n=171), respectively. Concerning the adoption of disordered eating behaviors, approximately one-fifth of the women exhibited at least one such behavior, while 3.1% (n=28) demonstrated two or more. Among the UWCP, the most prevalent practice was skipping meals or eating very little, with a prevalence of 15.8% (n=117) among respondents. The mean value for emotional social support was 16.0 (SD=3.9).

**Table 1.** Characteristics of the study population. Duque de Caxias (RJ), Brazil, 2010 (n=847).

	n	%	95%CI
Woman’s age (years)			
<20	24	1.0	0.5-2.0
20-29	245	20.8	16.9-25.3
30-39	251	34.1	28.4-40.2
40-49	199	19.1	15.9-22.8
≥50	128	25.0	18.7-32.7
Race/ethnicity			
White	269	36.6	29.4-44.6
Black/Mixed/Yellow/Indigenous	571	63.4	55.4-70.6
Living with partner			
Yes	739	89.4	84.8-92.7
No	102	10.6	7.2-15.2
Economic classification			
B	50	4.2	2.7-6.5
C	497	67.2	60.4-73.4
D-E	244	28.6	22.2-35.9
Psychological intimate partner violence			
No	308	39.2	31.7-47.2
Yes	526	60.9	52.8-68.3
Physical intimate partner violence			
No	662	83.2	76.2-88.5
Yes	171	16.8	11.5-23.8
Intimate partner violence			
None	297	38.1	30.6-46.1
Only psychological violence	367	45.1	38.8-51.5
Only physical violence	12	1.1	0.4-2.9
Both (psychological and physical violence)	159	15.7	10.6-22.7
Fasting			
Not once	718	84.2	79.5-88.0
At least once	117	15.8	11.9-20.5
Vomiting			
Not once	821	97.5	94.1-98.9
At least once	14	2.5	1.0-5.9
Laxatives			
Not once	799	95.8	93.7-97.2
At least once	42	4.2	2.8-6.3
Diuretics			
Not once	804	94.9	90.9-97.2
At least once	36	5.1	2.8-9.1
Unhealthy weight control practices			
None	655	76.2	69.4-82.0
Just one	147	20.7	15.5-27.0
Two or more	28	3.1	2.0-4.8

CI: confidence interval.

Table 2 shows a higher proportion, but not statistically significant, of women aged 30-39 years, who self-declared as non-white, belonged to economic stratum C, and were living with a partner, among those exposed to psychological and physical IPV, as well as among those engaging in UWCP.

**Table 2.** Intimate partner violence and unhealthy weight control practices stratified by age, race/ethnicity, living with partner, and economic classification. Duque de Caxias (RJ), Brazil, 2010 (n=847).

	Exposed to psychological IPV			Exposed to physical IPV			Adopted unhealthy weight control practices*		
	%	95%CI	p-value	%	95%CI	p-value	%	95%CI	p-value
Woman’s age (years)									
<20	1.4	0.7-3.0	0.0550	1.6	0.8-3.6	0.4049	0.4	0.1-1.3	0.4764
20-29	27.1	21.5-33.4		27.9	17.0-42.2		26.5	16.2-40.2	
30-39	37.5	31.1-44.4		42.8	29.4-57.3		39.6	30.8-49.1	
40-49	19.4	14.8-25.0		14.7	8.4-24.5		16.2	10.7-23.9	
≥50	14.6	11.0-19.1		13.0	6.8-23.4		17.3	9.2-30.2	
Race/ethnicity^†^									
White	35.8	28.3-44.1	0.4039	40.9	27.8-55.5	0.6606	28.9	19.7-43.7	0.1908
Black/Mixed	64.2	55.9-71.7		59.1	44.5-72.2		71.1	59.7-80.3	
Living with a partner									
Yes	88.0	81.5-92.4	0.2317	87.0	68.3-95.4	0.6201	87.7	77.5-93.6	0.5568
No	12.0	7.6-18.5		13.0	4.6-31.7		12.3	6.4-22.5	
Economic classification									
B	4.7	2.6-8.4	0.1075	0.8	0.2-3.1	0.1181	3.4	1.3-8.8	0.2941
C	61.6	52.8-69.7		61.9	44.9-76.3		73.5	60.6-83.3	
D-E	33.7	25.5-43.0		37.3	22.9-54.4		23.1	13.7-36.3	

IPV: intimate partner violence; CI: confidence interval.

*Woman who has used any of the specified methods at least once in the 6 months before the interview: laxatives, diuretics, vomiting, or fasting; ^†^For this analysis, yellow (Asian descent; n=29) and indigenous (n=5) are excluded.

The adjusted logistic regression models (Table 3) indicate that exposure to both psychological and physical IPV increases the likelihood of women’s self-induced vomiting as a means of attempting to control or lose weight. The strength of this association was greater in women exposed to physical violence (odds ratio [OR] 1.75; 95% confidence interval [CI] 1.42-2.16) than psychological violence (OR 1.36; 95%CI 1.10-1.69). The probability of women engaging in dietary restriction or skipping meals with the objective of weight control or weight loss was also elevated among those who reported exposure to physical IPV (OR 1.28, 95%CI 1.05-1.57). No significant evidence was found to suggest an association between IPV and other types of UWCP.

**Table 3.** Crude and adjusted analysis of the relationship between exposure to intimate partner violence and unhealthy weight control practices (separately). Duque de Caxias (RJ), Brazil, 2010 (n=847).

	Psychological intimate partner violence			
	Unadjusted		Adjusted*	
	OR	(95%CI)	OR	(95%CI)
Laxatives	1.10	(0.86-1.40)	1.16	(0.88-1.53)
Diuretics	0.73	(0.53-1.01)	0.76	(0.52-1.10)
Vomiting	1.38	(1.19-1.61)	1.36	(1.10-1.69)
Fasting	1.13	(0.95-1.34)	1.08	(0.89-1.32)
	**Physical intimate partner violence**			
	**Unadjusted**		Adjusted*	
	**OR**	**(95%CI)**	**OR**	**(95%CI)**
Laxatives	1.14	(0.90-1.45)	1.21	(0.97-1.52)
Diuretics	0.96	(0.59-1.58)	1.06	(0.62-1.81)
Vomiting	1.56	(1.24-1.95)	1.75	(1.42-2.16)
Fasting	1.28	(1.04-1.57)	1.28	(1.05-1.57)

CI: confidence interval.

*Adjusted for woman’s age, race/ethnicity, living with a partner, economic classification, and emotional social support.

Furthermore, the logistic regression models indicated that exposure to physical IPV was associated with an increased likelihood of women adopting at least one UWCP, with a particularly pronounced effect observed for the adoption of two or more practices (OROne 1.26, 95%CI 1.07-1.49 vs. OR_2_ or more 1.54, 95%CI 1.16-2.03) (Table 4). No significant results were found between both forms of violence combined (psychological and physical) and UWCP (Table 4).

**Table 4.** Crude and adjusted analysis of the relationship between exposure to intimate partner violence and unhealthy weight control practices. Duque de Caxias (RJ), Brazil, 2010 (n=847).

Psychological IPV against women				
UWCP*	Unadjusted		Adjusted^†^	
	OR	(95%CI)	OR	(95%CI)
None	-	-	-	-
Only one	1.00	(0.83-1.20)	0.98	(0.81-1.19)
Two or more	1.23	(0.93-1.63)	1.24	(0.87-1.77)
**Physical IPV against women**				
UWCP*	**Unadjusted**		**Adjusted^†^**	
	**OR**	**(95%CI)**	**OR**	**(95%CI)**
None	-	-	-	-
Only one	1.24	(1.05-1.47)	1.26	(1.07-1.49)
Two or more	1.36	(1.08-1.70)	1.54	(1.16-2.03)
**Psychological and physical IPV against women**				
UWCP*	**Unadjusted**		**Adjusted^†^**	
	**OR**	**(95%CI)**	**OR**	**(95%CI)**
None	-	-	-	-
Only one	1.20	(0.93-1.56)	1.61	(0.77-3.39)
Two or more	1.29	(0.81-2.06)	2.45	(0.44-13.53)

UWCP: unhealthy weight control practices; IPV: intimate partner violence; CI: confidence interval.

*Unhealthy weight control practices included the use of at least one of the practices (laxatives, use of diuretics, self-induced vomiting, or fasting); ^†^Adjusted for woman’s age, race/ethnicity, living with a partner, economic classification, and emotional social support.

## DISCUSSION

As previously stated, findings align with those of prior studies conducted in high-income countries, indicating that women in Brazil who experience IPV are more likely to engage in UWCP, such as vomiting or skipping meals, compared to those who are not victims of IPV. Specifically, those who were victims of psychological IPV were more likely to engage in self-induced vomiting for weight control purposes than those who were not exposed to such violence. Similarly, victims of physical IPV were more likely to engage in self-induced vomiting and to skip meals or eat very little for the same purpose than those who were not exposed to such violence.

Despite methodological differences across studies, including variations in sample characteristics (e.g., age, race, socioeconomic status) and differences in exposure and outcome measurement, the literature provides evidence of a positive association between exposure to violence and the adoption of these behaviors^11^ ^,^ ^13^ ^,^ ^19^ ^,^ ^20^ ^,^ ^33^ ^,^ ^34^.

Some hypotheses may provide a rationale for the plausibility of the relationship between the experience of IPV and the adoption of UWCP. The existing literature indicates that exposure to IPV has a detrimental impact on victims’ perception of their body image^35^ ^,^ ^36^ ^,^ ^37^. Additionally, numerous studies have indicated a significant correlation between body image dissatisfaction and the occurrence of disordered eating behaviors^38^ ^,^ ^39^. This relationship may arise directly from appearance-related insults by the abuser, which may result in a negative self-evaluation, or from the perceived discrepancy between one’s body and socially imposed beauty standards. Such factors can result in the adoption of compensatory behaviors, such as extreme dieting or other unhealthy eating practices, in an attempt to improve one’s appearance.

An additional potential explanation is that self-induced vomiting or skipping meals as weight control strategies may be poorly regulated methods of alleviating and coping with the emotional distress associated with exposure to IPV. This is consistent with the broader literature indicating that individuals with EDs frequently demonstrate challenges in adaptive emotion regulation, particularly in the acceptance of emotions. Maladaptive strategies, such as suppression, avoidance, and rumination, are frequently employed, which can serve to perpetuate the habit of food restriction as a daily coping mechanism^40^ ^,^ ^41^ ^,^ ^42^ ^,^ ^43^ ^,^ ^44^ ^,^ ^45^. As evidenced by meta-analyses conducted by Leppanen et al.^45^, difficulties in accepting emotions are closely associated with ED symptoms. Some individuals may resort to UWCP as a way to escape and avoid unwanted emotions. A substantial body of evidence indicates that prolonged exposure to violence, particularly IPV, is a significant contributing factor to the development and exacerbation of anxiety and depression^46^ ^,^ ^47^ ^,^ ^48^. These emotional disorders are strongly linked to the occurrence and progression of EDs^49^ ^,^ ^50^ ^,^ ^51^. Women who have experienced IPV may engage in food-related behaviors, including both restriction and binge eating, as maladaptive coping mechanisms to manage emotional distress. Furthermore, these behaviors may be a projection of the painful experience onto the body or a form of corporal punishment due to victimization^42^ ^,^ ^52^.

Another potential pathway that could explain the relationship between UWCP and IPV involves the chronic activation of the hypothalamic-pituitary-adrenal axis in response to stress. This is observed in women exposed to prolonged stressors, which can lead to elevated cortisol levels. Cortisol is a key hormone in stress regulation^53^ ^,^ ^54^ ^,^ ^55^. An increase in cortisol is directly associated with alterations in eating patterns, which may either enhance or suppress appetite, contributing to behaviors such as binge eating or loss of appetite^56^ ^,^ ^57^. Furthermore, elevated cortisol levels have been demonstrated to negatively impact metabolic processes, leading to an accumulation of adipose tissue, particularly in the abdominal region^57^ ^,^ ^58^. This increase in body fat can exacerbate body image dissatisfaction, thereby creating a vicious cycle that reinforces restrictive eating behaviors and further aggravates the metabolic impact of stress.

It is important to consider the limitations and strengths of this study when interpreting its findings. First, as this is a cross-sectional study, it does not allow for the well-establishment of the temporality of causal relationships. However, the temporal windows used for IPV (past 12 months) and UWCP (past 6 months) suggest a plausible sequence in which IPV precedes the adoption of UWCP. Nevertheless, given the chronic nature of IPV, it is reasonable to consider that exposure to IPV may lead to UWCP, rather than the reverse. The use of a single-question measure for UWCP, rather than a comprehensive, validated scale. The lack of a validated scale may have affected the accuracy of the results obtained.

Another limitation is the timing of data collection, which occurred over a decade ago. During the period under review, changes may have occurred in the prevalence of the issues analyzed as a result of developments in the prevention of IPV and the impact of the global COVID-19 pandemic. The pandemic was associated with an increase in IPV prevalence^59^ ^,^ ^60^ ^,^ ^61^ and contributed to changes in eating behaviors during this period^62^ ^,^ ^63^. However, it is important to highlight that our motivation for using data collected some time ago stems from the fact that, despite the ongoing global public health challenge posed by harmful weight control methods, there are few studies in low- and middle-income countries that incorporate psychosocial aspects, particularly victimization, using a validated questionnaire to identify the problem. Additionally, this study did not test for mediation effects and did not include some potentially important covariates - such as mental health status, body image dissatisfaction, body mass index, and physical activity - as potential confounders. All of these limitations should be addressed in future research.

The main strength of this study is its concentration on the core theme. To the best of our knowledge, this study is the inaugural investigation to assess the adoption of unhealthy weight control practices among adult women exposed to IPV and residing in a middle-income country. Furthermore, the utilization of a validated instrument to measure IPV serves to minimize the potential for misclassification errors, thereby enhancing the robustness of the findings. The results of this study offer significant insights into the relationship between IPV and the adoption of UWCP in adult women, which may prove valuable in guiding the design of future research in this area.

The findings indicated a relationship between IPV against women and the adoption of UWCP. Women victims of physical and psychological IPV were more likely to engage in harmful eating behaviors, such as vomiting and skipping meals with the intention of controlling or losing weight. These findings highlight the need for a comprehensive approach to care, emphasizing that health professionals - especially nutritionists, endocrinologists, and psychiatrists - must be aware of this relationship and be attentive to addressing the issue of disordered eating behaviors in patients who have suffered domestic violence. In addition, awareness needs to be raised about the importance of preventing domestic violence and the impact it can have on many aspects of victims’ lives, including their eating and health.
