# Supplementary material for: Intimate partner violence against women and the adoption of unhealthy weight control practices
Source: Rev Bras Epidemiol. 2025 Sep 19;28:e250048. doi: 10.1590/1980-549720250048 (PMC12448659; doi:10.1590/1980-549720250048)
Supplement: Supplementary file 1 [file 1980-5497-rbepid-28-e250048-supp.pdf]

Supplemental Material 1: Missing data for the variable of the study population.

Duque de Caxias, Rio de Janeiro, Brazil, 2010. (n=847)

	**n**	**% missing**
Woman's age (years)	10	1.2
Race/ethnicity	7	0.8
Living with a partner	6	0.7
Economic classification	56	6.6
Psychological intimate partner violence	13	1.5
Physical intimate partner violence	14	1.7
Intimate partner violence	3	0.6
Fasting	12	1.4
Vomiting	12	1.4
Laxatives	6	0.7
Diuretics	7	0.8
Unhealthy Weight Control Practices	17	2.0
**TOTAL**	97	11.5

Supplementary Material 2

BACKGROUND INFORMATION ON THE SURVEY CONDUCTED CAMPOS ELÍSEOS, THE SECOND DISTRICT OF DUQUE DE CAXIAS, RIO DE JANEIRO, BRAZIL

1. Survey Background

When the sample for this research was planned, the 2010 Brazilian Census (IBGE) was not yet available. The geographic operational base (territorial divisions and grid of sectors was still the same as in the 2000 Demographic Census, given that Duque de Caxias, due to its large population size, had not been included in the 2007 Population Count.

Another issue addressed concerned the field mapping of selected sectors. In the previous survey conducted in 2005, this process took a long time to complete in the field, type, and process. Regarding sample size, the 2005 survey sample was considered adequate for carrying out the main analyses. These aspects guided the adaptations made to the 2005 sampling design for the 2010 survey. The main changes implemented were:

(1) ot selecting new sectors, keeping the same 75 sectors from 2005, for which household listings were already available;

(2) replacing the full household listing with a simple update of the list of private households and using inverse sampling to select households with children; and

(3) not selecting one adult at random in households with children or adolescents, but rather interviewing their main caregiver (usually the mother).

2. Study population

The study population corresponds to all the families living in private households in Campos Elíseos, the second district of the municipality of Duque de Caxias, state of Rio de Janeiro, on the reference dates of each survey (01/08/2010).

In the 2000 Demographic Census, the Campos Elíseos district had a resident population of 243,767 people, of whom 243,013 (99.7%) lived in the 67,865 private households that existed at the time. For the census operation, the district was divided into 322 census sectors.

In the 2010 Demographic Census, the same area had a resident population of 290,762 inhabitants, of which 290,612 (99.9%) lived in the 89,834 private homes that existed at the time. For the census operation, the district was divided into 388 census sectors.

The data above shows that the research population is practically the same as the population living in the Campos Elíseos district on the dates considered, since the population living in collective (non-private) homes is almost zero in the district.

3. Sample Design

The 2010 SANDUC sample followed a three-stage cluster design (sector, household, and person). The same 75 were considered as in the 2005 SANDUC survey sample, and therefore their probabilities of inclusion in the sample remained unchanged for 2010.

When selecting the 15 households in each sector, a double inverse sampling procedure (Haldane, 1945) was adopted to interview up to eight households with children and the complement of fifteen of any other type (without children, and with or without adolescents).

This process was supported by spreadsheets - one for each sector - to facilitate the selection of households and people. As the quick count (based on the lists from the 2005

sweep) was carried out, the total number of households and households with children were entered into the spreadsheets. Depending on the formulas programmed into the spreadsheet, the order numbers of the households (with children and without children) to be visited were automatically indicated in the selection tables. The spreadsheet formulas established a random sequence of household order numbers in the two selection tables.

Initially, households with children were visited sequentially, respecting the random order of the visits, until eight interviews were obtained or the number of households with children was exhausted. At each visit, the result of the interview was recorded (1- refusal; 2- demolished; 3- closed; 4- vacant or in occasional use; 5- ineligible for the stratum; and 6- interview carried out).

Next, the total number of interviews carried out in households with children was entered to determine the number of interviews to be carried out in households without children, i.e., the complement to 15. Then the same process of visits began, observing the order established in the table of households without children.

This procedure is known as inverse sampling because, instead of determining the number of households in the sample, to try to interview everyone (always obtaining some non-response), it fixes the number of interviews carried out. In this sense, inverse sampling can be defined as the method that consists of checking how many units need to be observed (n) to obtain k successes (interviews carried out or units of interest).

This design was proposed to simplify the household selection procedures while ensuring a sufficient number of households with children to enable the planned analyses to be carried out.

In the selection of people, the only change envisaged would be the inclusion of the mother of (or guardian of) the selected child or adolescent, and the fact that the food security questionnaire would be filled in by the person in charge of the household.

4. Sample size

Sample size was calculated based on an estimated extreme poverty prevalence (P) of 15%, with a relative error of 14%, corresponding to a maximum absolute error of 2.1% (= 14% × 15%), at a 5% significance level. With these parameters and expression (1), we arrive at a simple random sample size (n_{AAS}) of 1,111 households.

$$n_{AAS} = \frac{1,96^2 \times V_y^2}{k_r^2} = \frac{1,96^2 \times 5,7}{0,14^2} = 1.111$$

Where:

1,96 corresponds to the abscissa associated with the 5% significance level;

$V_y^2 = \frac{P(1-P)}{P^2} = \frac{1-P}{P} = \frac{0,85}{0,15} = 5,7$ is the variance of the proportion estimator (P=15%);

K_r is the relative error of 14%, which, multiplied by the proportion of 15% leads to a maximum error in the estimation of P of 2.1%.

5. Probabilistic sampling scheme and calculation of the natural weight of the design

The probability of inclusion in the sample of the ith sector, represented by $P(S_i)$, is given by the expression:

$$P(S_i) = \frac{n_s \times DP_i}{DP}$$

Where:

n_s is the sample size of sectors (= 75);

DP_i	is the number of private households in sector i in the 2000 Demographic Census; and
$DP = \sum_i DP_i$	is the total number of private households in the district of Campos Elíseos.

The probability of including households depends on the type of household. If we represent the household with a child by DC and the household without children by DS, the probability of inclusion of the jth household in the ith sector is given by expressions (1) and (2).

$$P(DC_{ij} \mid S_i) = \frac{vc_i}{N_i} \times \frac{ac_i}{vc_i} \times \frac{nc_i - 1}{ac_i - 1} \quad \text{or} \tag{1}$$

$$P(DS_{ij} \mid S_i) = \frac{vs_i}{N_i} \times \frac{as_i}{vs_i} \times \frac{ns_i - 1}{as_i - 1} \quad \text{where:} \tag{2}$$

vc_i and vs_i	are respectively the number of households with children and without children visited in sector i;
ac_i and as_i	are respectively the number of households with children and without children belonging to the target population in sector i;
nc_i and ns_i	are respectively the number of households with children and without children actually interviewed in sector i.

The expressions (1) and (2) are composed of three ratios which indicate three conditional probabilities: the first is that the household is visited, given that the sector has been selected; the second is that the household belongs to the target population given that it has been visited; and the third is that the household is one of the first n to accept the interview given that it belongs to the target population. The third ratio has the "- 1" in

the numerator and denominator due to the loss of the degree of freedom related to the stopping rule: The last household to accept the interview is also the last in the target population that was visited.

6. Calibration of natural weights and calculation of calibrated weights of households and people

The expression of the weights of people was predominantly based on the number of households (except in the last ratio of the expression below, where sex and age group within the household are considered). Thus, it can be expected that the weights and people will not adequately estimate the population by sex and age.

$$W_{ihjG} = \frac{DP}{n_s \times DP_i} \times \frac{N_{ih}}{n_{ih}} \times \frac{N_{ihjG}}{n_{ihjG}}$$

This is common in household surveys, where the weights cannot accurately estimate population totals by sex and age group, only household totals.

In fact, this has been the main justification for the calibration of household weights (Silva, 2004): consistency between estimates from the household sample survey and population totals known from other sources (in this case IBGE population projections).

The population totals used are shown in Table 1 and were obtained using the same techniques applied by the IBGE in its population estimates (IBGE, 2008).

The basic idea of calibration is to estimate (by regression) calibration factors, which multiply the natural weights of the design to determine calibrated weights that have the property of minimizing the differences between the estimates they produce and the known population totals for a set of auxiliary calibration variables.

In this case, the auxiliary calibration variables were sex, age group and total households, whose values are shown in Table 1.

Table 1 - Resident population in the Campos Elyseos district on 1/7/2005, by sex, according to age group

Age group	Population	
	Man	Woman
Total	129,696	135,269
18 - 35 months	5,686	5,511
03 - 11 years	27,429	26,650
12 - 17 years	17,649	17,896
19 - 39 years	46,695	48,763
40 - 59 years	23,971	26,294
60 years or more	8,266	10,155

The total number of households used in the calibration was 73,767

The most common calibration method in household surveys is the Household Integrated Weighting System, which makes it possible to obtain household weights that produce estimates that coincide (or minimize differences) simultaneously with the totals of households and people obtained from an exogenous source, as described in Vasconcellos et al. (2005).

Using this method, calibrated household weights were obtained, as indicated in the expression below. The expression has been simplified to avoid perverse notation for the reader. If you wish, it has been made more precise.

$$W_{ihP}^{c} = W_{ihP} \times FC_{P}$$

Where:

W_{ih}^{c} is the calibrated weight of households in stratum h of sector i, for combination P of the values of the calibration variables; and

FC_{P} is the calibration factor calculated by regression for combination P of the values of the auxiliary calibration variables.
